# A highly sensitive method for the detection of recombinant PERV-A/C env RNA using next generation sequencing technologies

**DOI:** 10.1038/s41598-020-78890-2

**Published:** 2020-12-14

**Authors:** Ken Kono, Kiyoko Kataoka, Yuzhe Yuan, Keisuke Yusa, Kazuhisa Uchida, Yoji Sato

**Affiliations:** 1 Division of Cell-Based Therapeutic Products, National Institute of Health Sciences, 3-25-26 Tonomachi, Kawasaki Ward, Kawasaki City, Kanagawa 210-9501 Japan; 2 Graduate School of Science, Technology and Innovation, Kobe University, Hyogo, Japan; 3 Department of Translational Pharmaceutical Sciences, Graduate School of Pharmaceutical Sciences, Kyushu University, Fukuoka, Japan; 4 Department of Cellular and Gene Therapy Products, Graduate School of Pharmaceutical Sciences, Osaka University, Osaka, Japan

**Keywords:** Next-generation sequencing, Bioinformatics

## Abstract

Several xenogenic cell-based therapeutic products are currently under development around the world for the treatment of human diseases. Porcine islet cell products for treating human diabetes are a typical example. Since porcine cells possess endogenous retrovirus (PERV), which can replicate in human cells in vitro, the potential transmission of PERV has raised concerns in the development of these products. Four subgroups of infectious PERV have been identified, namely PERV-A, -B, -C, and recombinant PERV-A/C. Among them, PERV-A/C shows a high titre and there was a paper reported that an incidence of PERV-A/C viremia was increased in diseased pigs; thus, it would be important to monitor the emergence of PERV-A/C after transplantation of porcine products. In this study, we developed a highly sensitive method for the detection of PERV-A/C using next generation sequencing (NGS) technologies. A model PERV-C spiked with various doses of PERV-A/C were amplified by RT-PCR and the amplicons were analysed by NGS. We found that the NGS analysis allowed the detection of PERV-A/C at the abundance ratios of 1% and 0.1% with true positive rates of 100% and 57%, respectively, indicating that it would be useful for the rapid detection of PERV-A/C emergence after transplantation of porcine products.

## Introduction

While the development of superior immunosuppressive drugs and improved medical treatments has made organ transplantation an established medical practice, the chronic shortage of human donor organs remains a problem. The Global Observatory on Donation and Transplantation reported that 139,024 organ transplants were performed all over the world in 2017, but this only covers 10% of the global need^1^. In addition to artificial organs, xenogenic cell-based therapeutic products have been expected to become a breakthrough of this problem^2–4^, and several products, such as porcine islet cell products for treating human diabetes, are currently under development around the world^5–7^.

One of the major concerns in xenogenic cell-based therapeutic products is the transmission of pathogens carried by donor animals to human recipients^8,9^. In the case of pigs, which have been well-studied donors for xenotransplantation, it has been demonstrated that most of known microorganisms can be eliminated by performing a caesarean section and by controlling the breeding environment^5,9^, with the sole exception of porcine endogenous retroviruses (PERV)^10,11^. PERV infected porcine germ cells in ancient times, and thus all porcine cells possess PERV proviruses in their genome^12–14^. Four subgroups of PERV have been identified, PERV-A, -B, -C, and recombinant PERV-A/C. Both PERV-A and PERV-B are capable of infecting human cells, whereas PERV-C is not able to infect human cells^15,16^. PERV-A/C is a recombinant PERV-C in which a portion of the *env* has been recombined with that of PERV-A. Although the genome sequence of PERV-A/C has been shown to be almost identical to that of PERV-C^17^, the recombinant virus can replicate in human cells^18,19^. Since PERV-A/C was originally detected after cocultivation of porcine cells with human cells^18,20^ and has not been found in the porcine germ line^21^, the recombination was considered to have happened in human cells. However, PERV-A/C was found in somatic cells of miniature swine, indicating that PERV-A/C could also arise in porcine cells alone without human cell infection^22–25^. Because PERV-A/C recombinants replicate rapidly in human cells *in vitro*^18–20^ and an incidence of PERV-A/C viremia is increased in diseased swine^26^, it would be important to monitor for the appearance of PERV-A/C after xenotransplantation of porcine cell-based therapeutic products^19^, even though PERV infection in humans exposed to living porcine cells has not been reported thus far^7,27^.

The PCR assay is considered to be the most sensitive and rapid method for the detection of known microorganisms and there are reliable primers for detecting PERV-A/C recombinants^22,28^. However, since the recombination sites between PERV-A and PERV-C are not fixed^18,20,23^ and these viral genome sequences are highly identical, there is a concern about false positive and negative. Therefore, examining the sequence of PCR-amplified products is thought to be more reliable in the case of detecting the recombinants. There are 2 classical methods based on Sanger sequencing used in detecting mutations: direct sequencing, in which the PCR-amplified target DNA region is read directly, and sequencing after cloning, in which the amplified DNA is incorporated into a plasmid, cloned in *E. coli*, and then the inserted DNA of the plasmid is read. Although the direct sequencing method is simple, it has the disadvantage of low sensitivity because only the majority sequence can be read. On the other hand, the sequencing after cloning requires an increased number of clones for increased sensitivity, which is laborious and time-consuming. Amplicon sequencing (Amplicon-Seq) refers to deep sequencing of PCR products using next generation sequencing (NGS) technologies, and is performed for various applications, such as to detect somatic mutations in one or several exons and introns^29^ or to explore microorganisms in samples by sequencing the 16S rRNA^30^. It also has been used to detect minor variants of viral populations^31–33^. In this study, we performed Amplicon-Seq analysis using the primers that amplify the entire env region of PERV-C and PERV-A/C and characterized the ability of this analysis to detect the presence of PERV-A/C recombinants.

## Results

### Data processing

**Figure 1:**
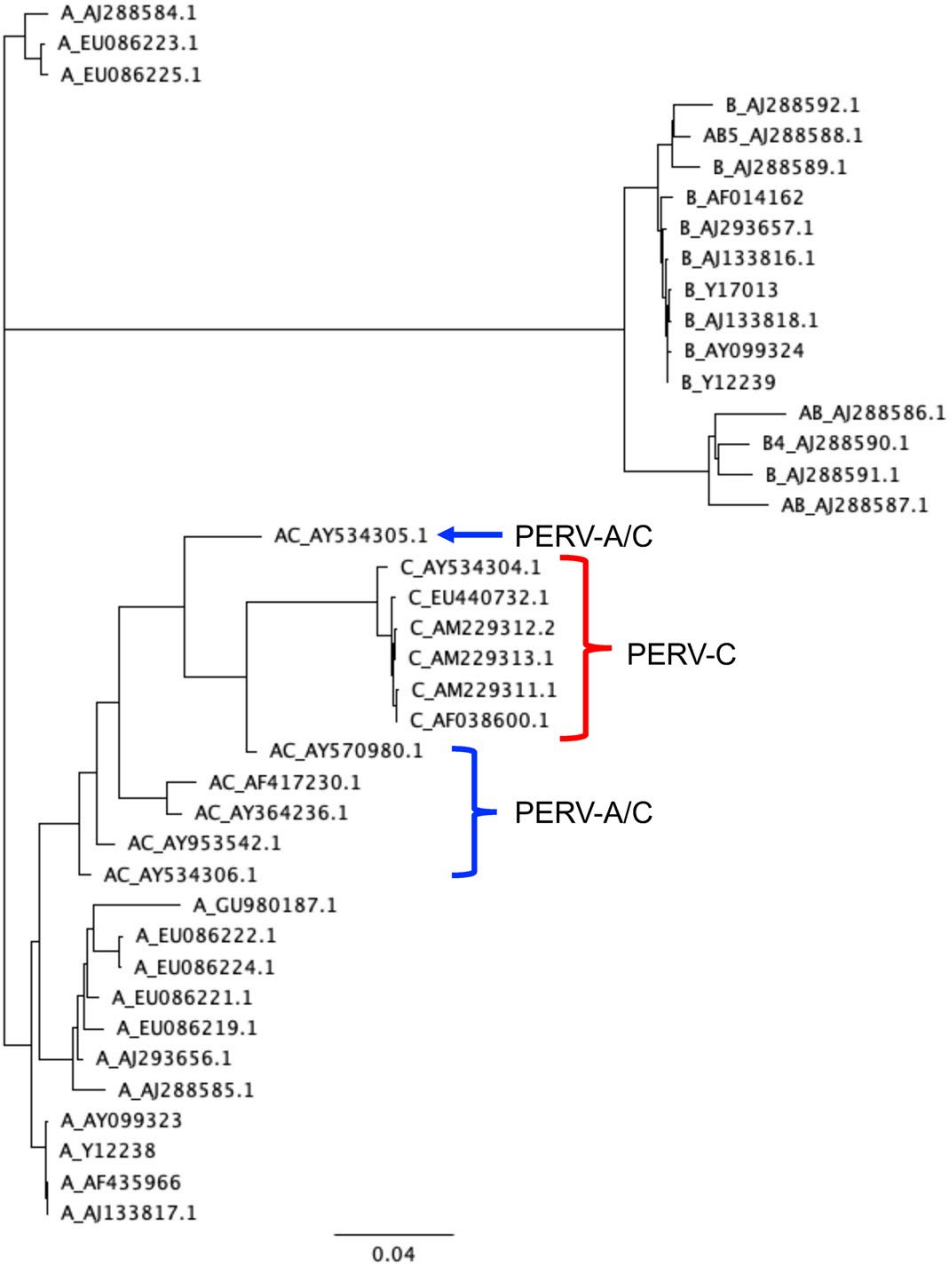
A phylogenetic tree of PERV subgroups. PERV *env* sequences obtained from the NCBI GenBank were multiple aligned, and obtained alignment data were used to create the phylogenetic tree using the neighbor-joining method.

To determine PERV-A/C recombinants, a phylogenetic tree was created from the multiple alignment of the NGS read sequences and PERV *env* sequences obtained from the NCBI GenBank, with NGS reads being classified by whether or not the read was in the same cluster as previously reported PERV-A/C *env* sequences (Fig. 1). The RNA sequences of the *env* of PERV-C (AF038600.1) and PERV-A/C (13653)^23^ synthesized by in vitro transcription reaction were used as model viruses, and analytic samples were prepared by mixing PERV-C and PERV-A/C at ratios of 1 to 0 (negative control), 1 to 0.1, 1 to 0.01, and 1 to 0.001, which were referred to as "0% PERV-A/C", "10% PERV-A/C", "1% PERV-A/C", and "0.1% PERV-A/C", respectively. The entire *env* region was amplified by reverse transcription polymerase chain reaction (RT-PCR), and the sequence of the amplified products, which were about 2 kbp, was determined by PacBio single molecule, real-time (SMRT) technology. The primers used in this reaction amplified neither PERV-A nor PERV-B. In our preliminary experiment, RT-PCRs using PERV-A&C-For and PERV-C-Rev as primers also showed no fragment amplification from total mRNA of porcine cell lines PKF (IFO50422), PKR (IFO50423), PKS (IFO50421), and LLC-PK1 (JCRB0060) (Supplementary Fig. S1 online), indicating not only the absence of PERV-C and A/C in the cell lines but also the specificity of the primers. We used the PacBio RS II as the NGS platform, because it is suitable for long-read sequencing. Raw data were initially processed through the PacBio SMRT portal and filtered for a minimum of 6 passes expected for accuracy of 99.9%^34^.

Six independent analyses offered approximately 23,000 to 44,000 sequence reads from each sample (Table 1). As the amount of the reads was too large for multiple alignment analysis, we did some processing to reduce the data. First, reads were filtered for a length of 1500 bp or longer, resulting in a reduction to approximately 7,400 to 36,000. A homology analysis was then performed to further reduce the amount of data. To determine which PERV sequence would be suitable for the query, we first performed the homology analysis among PERV-C and PERV-A/C subgroups obtained from the NCBI GenBank (Table 2). Both global and local alignments were conducted for homology analysis, and both analyses showed that PERV-C was highly conserved among the subgroups, indicating that PERV-C was suitable for the query sequence. Next, a homology analysis was performed between PERV-C and PERV-A/C (Table 3). The identity between PERV-C and PERV-A/C was 83 to 93% by global alignment and 70 to 93% by local alignment. Therefore, we used PERV-C (AF038600.1) as the query and extracted the NGS reads with 80 to 94% identity of the global alignment or 70 to 94% identity of the local alignment. The read number after global alignment was under 1,000, which was manageable for use in the multiple alignment analysis. On the other hand, because some of the data after local alignment contained more than 1000 reads, these data were divided into several files of under 1,000 reads each.

**Table 1:** Summary of the number of reads from the NGS analysis.

				Global	Global	Local	Local
Exp ID	A/C:C Ratio (%)	total	over1500	identity 80–94	AC reads	identity 70–94	AC reads
RSII 1	0%	22,893	15,535	628	0	1235	0
	10%	29,721	22,542	624	> 100	1405	> 100
	1%	33,170	24,967	650	8	1451	20
	0.1%	35,492	28,199	580	0	1389	1
RSII 2	0%	26,455	21,596	444	0	1062	0
	10%	23,713	19,591	605	> 100	1404	> 100
	1%	32,277	28,214	297	15	672	29
	0.1%	31,075	25,543	387	0	814	2
RSII 3	0%	40,171	31,706	443	0	1038	0
	10%	44,241	36,269	986	> 100	2123	> 100
	1%	39,856	33,456	434	30	1039	49
	0.1%	44,187	34,973	528	1	1241	1
RSII 4	0%	27,777	10,305	301	0	794	0
	10%	42,282	27,125	1166	> 100	2480	> 100
	1%	29,364	16,804	438	21	1056	32
	0.1%	26,039	13,902	367	0	898	0
RSII 5	0%	30,963	15,400	606	0	1410	0
	10%	27,154	18,303	699	> 100	1620	> 100
	1%	36,550	21,307	741	20	1761	30
	0.1%	32,212	17,961	486	0	1076	0
RSII 6	0%	31,988	12,880	708	0	1665	0
	10%	31,782	12,014	863	> 100	1978	> 100
	1%	30,231	7,359	510	2	1312	6
	0.1%	31,885	12,916	684	1	1586	1

**Table 2:** Identity (%) among PERV-C and PERV-A/C subgroups by global and local alignments.

PERV-C Global_Alignment	C_AF038600.1	C_AM229311.1	C_AM229312.2	C_AM229313.1	C_AY534304.1	C_EU440732.1
C_AF038600.1	*					
C_AM229311.1	99	*				
C_AM229312.2	99	99	*			
C_AM229313.1	99	99	99	*		
C_AY534304.1	99	99	99	99	*	
C_EU440732.1	99	99	99	99	99	*

PERV-C Local_Alignment	C_AF038600.1	C_AM229311.1	C_AM229312.2	C_AM229313.1	C_AY534304.1	C_EU440732.1
C_AF038600.1	*					
C_AM229311.1	99	*				
C_AM229312.2	99	99	*			
C_AM229313.1	99	99	99	*		
C_AY534304.1	99	98	99	99	*	
C_EU440732.1	99	99	99	99	99	*

**Table 3:** Identity (%) between PERV-C and PERV-A/C by global and local alignments.

PERV-C *vs.* A/C Global_Alignment	AC_AF417230.1	AC_AY364236	AC_AY534305.1	AC_AY534306.1	AC_AY570980.1	AC_AY953542.1
C_AF038600.1	89	88	93	86	92	87
C_AM229311.1	86	85	93	83	91	84
C_AM229312.2	89	88	93	86	92	87
C_AM229313.1	89	88	93	86	92	87
C_AY534304.1	90	89	93	86	93	87
C_EU440732.1	90	88	93	86	93	87

PERV-C *vs.* A/C Local_Alignment	AC_AF417230.1	AC_AY364236	AC_AY534305.1	AC_AY534306.1	AC_AY570980.1	AC_AY953542.1
C_AF038600.1	79	79	93	75	87	76
C_AM229311.1	73	73	93	70	83	70
C_AM229312.2	79	79	93	75	86	76
C_AM229313.1	79	79	93	75	87	76
C_AY534304.1	79	79	93	75	87	76
C_EU440732.1	79	79	93	75	86	76

### Specificity and true positive rate of the analysis

Phylogenetic trees were created from the multiple alignment data. Based on these, we determined the spiked PERV-A/C recombinants by whether or not reads were in the same cluster as previously reported PERV-A/C recombinants (see Supplementary Figs. S2, S3, S4, and S5 online). Since PERV-A/C was not detected in the 0% of samples in all experiments, the specificity of this analysis was regarded to be 100%. PERV-A/C at the abundance ratios of 10% and 1% was always detectable, regardless of the alignment method (Table 1). The number of PERV-A/C reads detected after local alignment was significantly more than that after global alignment (*p* < 0.003, two-way repeated measures analysis of variance and the Student–Newman–Keuls’s post-hoc test), indicating that local alignment was more suitable for this analysis. The mean number of the PERV-A/C reads detected in the 0.1% PERV-A/C samples was 0.33 and 0.83 using global and local alignments, respectively. Assuming that the PERV-A/C reads follow the Poisson distribution, the true positive rate of the 0.1% PERV-A/C samples was calculated to be 28% an 57%, when global and local alignments were applied, respectively (Table 4).

**Table 4:** True positive rates in detection of PERV-A/C intermingled with PERV-C.

Global_Alignment	AC Read number		
A/C:C Ratio (%)	λ (mean, n = 6)	P(k = 0)	P(k > 0)
0%	0.0	1.00 (TNR)	0.00 (FPR)
10%	> 100	0.00 (FNR)	1.00 (TPR)
1%	16.0	0.00 (FNR)	1.00 (TPR)
0.1%	0.33	0.72 (FNR)	0.28 (TPR)

Local_Alignment	AC Read number		
A/C:C Ratio (%)	λ (mean, n = 6)	P(k = 0)	P(k > 0)
0%	0.0	1.00 (TNR)	0.00 (FPR)
10%	> 100	0.00 (FNR)	1.00 (TPR)
1%	27.7	0.00 (FNR)	1.00 (TPR)
0.1%	0.83	0.43 (FNR)	0.57 (TPR)

TNR: true negative rate, FPR: false positive rate, FNR: false negative rate, TPR: true positive rate.

## Discussions

Although xenotransplantation has been expected to serve as a potential solution to the chronic donor shortage in transplantation, its application has not progressed well due to concerns regarding immunological rejection and transmission of infectious diseases. The problem of immunological rejection has been resolved in recent years with the development of superior immunosuppressive drugs and advances in gene editing technology^35,36^, but there is still much uncertainty about the transmission of infectious diseases caused by known or unknown microorganisms. The prediction of the transmission of animal viruses to humans from in vitro or in vivo studies is difficult, and thus it is important to monitor the infection in the actual transplanted patients. The Amplicon-Seq with the local alignment detected PERV-A/C recombinants at the abundance ratios of 10%, 1%, and 0.1% with true positive rates of 100%, 100%, and 57%, respectively (Table 4). Although highly sensitive PCR methods for the detection of PERV-A/C have already been established^22,28^, there is a possibility that they fail to detect the novel PERV-A/C which possess new recombination sites. Therefore, the Amplicon-Seq analysis in this study could be a complementary method for the early detection of PERV-A/C in patients after xenotransplantation of porcine cell-based therapeutic products.

The number of PERV-A/C reads derived from the 1% PERV-A/C samples after global alignment was significantly less than that after local alignment, and the 0.1% PERV-A/C samples showed the same trend. We examined the relationship between the sequence identity and the read number and found that there was a large peak at 40 to 49% in the global alignment (see Supplementary Fig. S6 online). This peak contained about half of total reads, suggesting that the PERV-A/C reads were lost there. Because global alignment compares the end to end sequences, reads which were not read at the full length could be scored as low identity. On the other hand, local alignment compares the most matched portion of the sequences, thus partial reads might have been unaffected by the analysis.

In this study, the Amplicon-Seq analysis using PacBio RS II showed that more than 100 recombinant reads were detected in the 10%, 6 to 49 reads in 1%, and 0 to 2 reads in 0.1% of the samples. The number of detected reads was roughly correlated with the concentration of the samples, suggesting that the first NGS data would need to be increased by a factor of 10 or more in order to detect the recombinants at a ratio of 0.01% or less in the samples. This increase in the obtained data would be possible by using other NGS equipment, such as PacBio Sequel I or II. On the other hand, the increase of data would lead to a heavy burden in analysis. Multiple alignment especially takes time to be calculated and thus it is important to reduce the number of reads before that in order to process the analysis as smoothly as possible using general computers. Sequence clustering, which sorts nucleotide or amino-acid sequences into clusters based on sequence similarity is used as one of the methods to reduce the NGS data^37,38^. Since the global alignment is used for clustering and the homology between PERV-C and PERV-A/C is less than 93%, clustering at 95% similarity or more might reduce the data without affecting the detection sensitivity.

Although PERV-A/C recombinants were detected in 1% of samples in 6 independent experiments, we were unable to detect them when we analysed low-purified RT-PCR products (see Supplementary Table S1 and Figure S7 online). This result indicated that it is important to set a quality standard of the band purity after electrophoresis to ensure the quality of this analysis. In addition, it is important to clarify the detection limit of the lowest PERV-A/C copy number. However, the primers used in this Amplicon-Seq analysis bind to not only PERV-A/C but also PERV-C, thus the PCR amplified products consist of both PERV-C and PERV-A/C. Since PERV-C mRNA is constantly expressed in cells which possess PERV-C proviruses in their genome, the only abundance ratios contribute to the sensitivity of the analysis.

Amplicon-Seq analysis in this study is assumed to be a complementary method for the detection of PERV-A/C in patients after xenotransplantation of porcine cell-based therapeutic products. Additionally, this approach could be applied not only as a detection method for the emergence of PERV-A/C in porcine organs, tissues, or cells intended for transplantation, but also an option to estimate the amount of other PERVs. There have been several attempts of developing pigs in which their PERVs were inactivated by using genome editing technologies^39,40^. However, the previous and most of ongoing porcine pancreatic islet products were derived from normal pigs^9,27^. Therefore, the importance of the analysis would continue in terms of the safety use and public health.

## Materials and methods

### Model viruses and generation of PERV env standard

To generate PERV-A *env* cDNA, total RNA was extracted from 293-PERV-PK-CIRCE (Public Health England)^13^ , which have been infected with both PERV-A and -B but not with PERV-C, using an RNeasy Mini Kit (Qiagen, Hilden, Germany) following the manufacturer’s instructions. RT reaction was conducted by using a ReverTra Ace qPCR RT Kit (TOYOBO, Osaka, Japan) and 5′- CTAGAGGTCAGTTTCTCCTTGGCTCAGAAGGCC-3′ (PERV-A-Rev) which binds to 3′ end region of PERV-A (Y12238) *env* as the gene specific primer. PCR was then performed by using KOD -Plus- Neo (TOYOBO) and primers of 5′- ATGCATCCCACGTTAAGCCGGCGCC-3′ (PERV-A-For), which binds to 5′ end region of PERV-A (Y12238) *env*, and PERV-A-Rev. The PCR cycling condition consisted of 2 min at 94 °C followed by 35 cycles of 98 °C for 10 s and 68 °C for 1 min (two-step PCR). Amplified products (1983 bp) were then cloned into the pTA2 vector (TOYOBO) and its nucleotide sequence was confirmed (pTA2/PERV-A env). To generate PERV-C *env* cDNA, total RNA was extracted from pig blood (SWINE BLOOD; Rockland, Limerick, Ireland), which was collected in accordance with relevant guidelines and regulations at the licensed Rockland facilities (USDA #23-R-184, NIH BPA#00008695, and NIH Assurance OPRR #A4062-01), using a NucleoSpin RNA Blood (TAKARA, Shiga, Japan) following the manufacturer’s instructions. RT reaction was conducted using a ReverTra Ace qPCR RT Kit (TOYOBO) and 5′-CTAGCGGCCAGCTTCCCTGCTAGACGG-3′ (PERV-C-Rev) which binds to 3′ end region of PERV-C (AF038600.1) and -A/C (AF417230.1) *env* as the gene specific primer. PCR was then performed by using KOD -Plus- Neo (TOYOBO) and primers of 5′-ATGCATCCCACGTTAAACCGGCGCCACCTCCCG-3′ (PERV-A&C-For), which binds to 5′ end region of PERV-A (Y12238), -C (AF038600.1), and -A/C (AF417230.1) *env*, and PERV-C-Rev. The PCR cycling condition consisted of 2 min at 94 °C followed by 35 cycles of 98 °C for 10 s and 68 °C for 1 min. The pig blood was purchased from a commercial source (Rockland) and no pigs have directly been handled as a part of this study. Amplified products (1917 bp) were then cloned into the pTA2 vector and its nucleotide sequence was confirmed (pTA2/PERV-C env).

PERV-A/C *env* in which a portion of the *env* has been recombined with that of PERV-A was generated by site-directed mutagenesis using the PCR-mediated overlap primer extension method^41^. The recombination site of the PERV-A/C was based on a previous report (PERV-AC env (13653))^23^. To generate the PERV-A/C *env*, 3 successive PCR reactions were performed using pTA2/PERV-A env and pTA2/PERV-C env as templates. The first PCR reaction used pTA2/PERV-A env as a template and PERV-A&C-For and 5′- GGTGTTGGTGGGATGGGGGAACCTTCCCTATGCAGGTGCCTTTTCC-3′ (underline; PERV-A (Y12238) env (1111–1131)) as primers, and the second used pTA2/PERV-C env as a template and 5′- GGAAAAGGCACCTGCATAGGGAAGGTTCCCCCATCCCACCAACACC-3′ (underline; PERV-C (AF038600.1) env (1068–1093)) and PERV-C-Rev as primers. The resultant first (1131 bp) and second (849 bp) fragments were used as templates in the third reaction with PERV-A&C-For and PERV-C-Rev as primers. The resultant products (1980 bp) were then cloned into the pTA2 vector and its nucleotide sequence was confirmed (pTA2/PERV-AC env (13653)). The sequence was nearly identical (98.8%) to that of the previous reported PERV-A/C (AF417230.1) *env* (see Supplementary Fig. S8 online).

To synthesize RNAs of PERV-C and PERV-A/C *env*, in vitro transcription reactions were performed using *Not* I-linearized pTA2/PERV-C env and pTA2/PERV-AC env (13653) as templates (RiboMAX Large Scale RNA Production System-T7, Promega, Madison, WI, USA). The synthesized RNAs were purified using PureYield RNA Midiprep System (Promega), and the purified RNAs were then used as model viruses.

### RT-PCR and cDNA purification

Briefly, 1 ng of PERV-C *env* RNA was mixed with serial diluted 0.1, 0.01, or 0.001 ng of PERV-A/C *env* RNA, and the mixtures were used as templates in RT-PCR reactions. RT reaction was conducted using a ReverTra Ace qPCR RT Kit (TOYOBO) and PERV-C-Rev which binds to 3′ end region of PERV-C (AF038600.1) and PERV-A/C (AF417230.1) as the gene specific primer. PCR was then performed by using KOD -Plus- Neo (TOYOBO) with primers of PERV-A&C-For which binds to 5′ end region of PERV-C (AF038600.1) and PERV-A/C (AF417230.1) and PERV-C-Rev. The PCR cycling condition consisted of 2 min at 94 °C followed by 35 cycles of 98 °C for 10 s and 68 °C for 1 min. Amplified products were then separated by agarose gel electrophoresis and products of 2kbp were purified using the FastGene Gel/PCR Extraction Kit (NIPPON Genetics, Tokyo, Japan). The purity of the products was confirmed using the Agilent 2100 Bioanalyzer (Agilent Technologies, Santa Clara, CA, USA).

### Sequencing

DNA samples were further purified using the AMPure XP (BECKMAN COULTER, Brea, CA, USA), blunt-ended, and ligated to SMRTbell adaptors using the SMRTBell Template Prep Kit 1.0 (Pacific Biosciences, Menlo Park, CA, USA) in order to construct SMRTbell libraries. One SMRT cell was used for each library and analysed using the PacBio RS II platform (Pacific Biosciences).

### Analysis pipeline

The Circular Consensus Sequence (CCS) reads from each SMRT cell were filtered for a minimum of 6 passes, and reads with under 1500 bp length were eliminated by the SeqKit (version 0.12.0)^42^. Global and local alignments were performed using the vsearch (version 2.14.2)^43^ and the GENETYX-Mac Ver.20 (GENETYX, Tokyo, Japan), respectively. After local alignment, using the SeqKit data containing over 1000 reads were divided into several files of less than 1000 reads each. Processed data were analysed by multiple alignment with PERV *env* sequences obtained from the NCBI GenBank (PERV-A: AF435966, AJ133817, AJ288584, AJ288585, AJ293656, AY099323, EU086219, EU086221, EU086222, EU086223, EU086224, EU086225, GU980187, Y12238; PERV-B: AF014162, AJ133816, AJ133818, AJ288586, AJ288587, AJ28588, AJ288589, AJ288590, AJ288591, AJ288592, AJ293657, AY099324, Y12239, Y17013; PERV-C: AF038600, AM229311, AM229312, AM229313, AY534304, EU440732; PERV-A/C: AF417230, AY364236, AY534305, AY534306, AY570980, AY953542), and multiple alignment data were used for creating phylogenetic trees using the Neighbor-Joining method^44^. Multiple alignment and construction of phylogenetic trees were performed using the Geneious Prime (Biomatters, Auckland, New Zealand).

### True positive rate of the analysis

The mean number of PERV-A/C reads detected in a single experiment was calculated (*λ*), and the probability of detecting *k* reads in a single experiment was calculated by the following probability mass function of the Poisson distribution:

$$P(k) = {e^{-\lambda}} {{\lambda}^{k}/{k!}}$$

where *k*! is the factorial of *k* and *e* is the Euler’s number (*e* = 2.71828…).

The true positive rate that is the probability of detecting one PERV-A/C read or more in a single experiment was given by 1 – *P* (*0*).

In this study, use of the term “true positive rate” was identical to “clinical/diagnostic sensitivity” as defined in ISO/TS 17822-1:2014^45^, whereas the term “sensitivity” was used in a broad sense.

### Data availability

The data that support the findings of this study are available from the corresponding author, YS, upon reasonable request.

## Supplementary Information

Supplementary Information.
