# Supplementary material for: A highly sensitive method for the detection of recombinant PERV-A/C env RNA using next generation sequencing technologies
Source: Sci Rep. 2020 Dec 14;10:21935. doi: 10.1038/s41598-020-78890-2 (PMC7736861; doi:10.1038/s41598-020-78890-2)
Supplement: Supplementary file 1 — Supplementary Information. [file 41598_2020_78890_MOESM1_ESM.pdf]

Supplementary Information

A highly sensitive method for the detection of recombinant PERV-A/C env RNA using next generation sequencing technologies

Ken Kono, Kiyoko Kataoka, Yuzhe Yuan, Keisuke Yusa, Kazuhisa Uchida, Yoji Sato

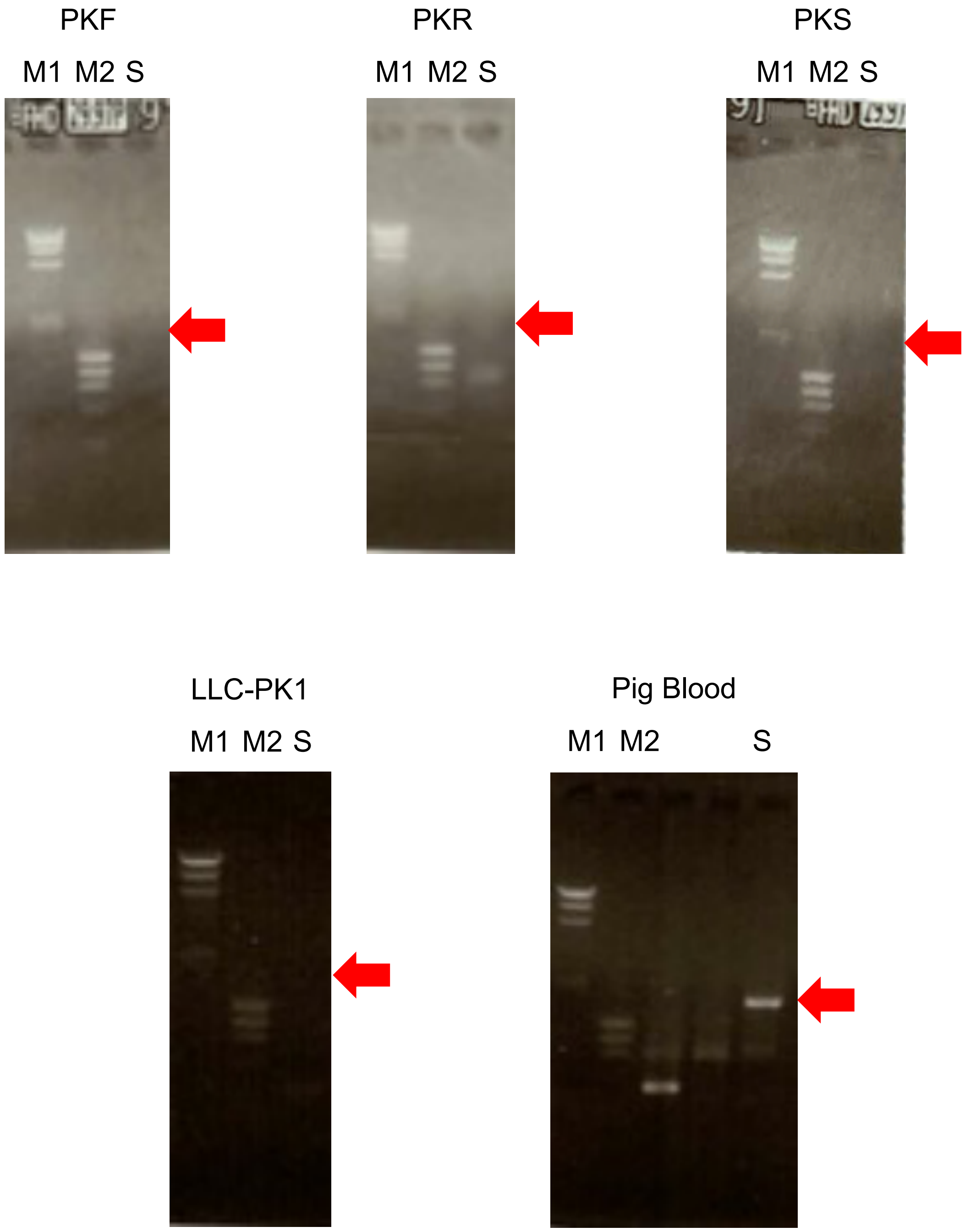

Figure S1. RT-PCRs using PERV-A&C-For and PERV-C-Rev as primers and total RNA of porcine cell lines, PKF (IFO50422), PKR (IFO50423), PKS (IFO50421), and LLC-PK1 (JCRB0060), and pig blood as templates. M1 and M2 denote the lanes of DNA makers, Lambda DNA-HindIII Digest and PhiX174 DNA-HaeIII Digest, respectively. S denotes the lane of the RT-PCR products. Red arrows indicate the predicted bands of amplified PERV-C env (2 kbp).

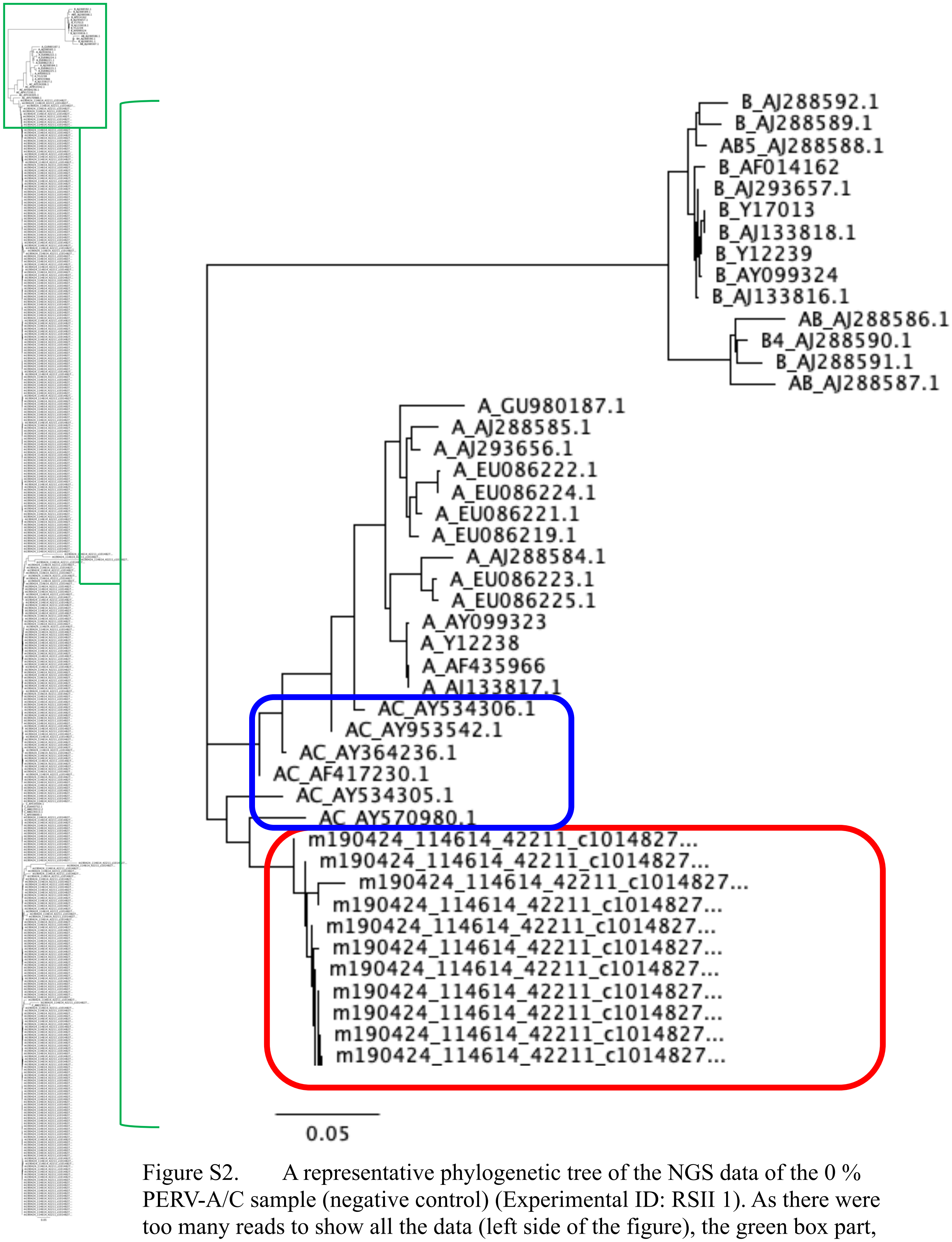

Figure S2. A representative phylogenetic tree of the NGS data of the 0 % PERV-A/C sample (negative control) (Experimental ID: RSII 1). As there were too many reads to show all the data (left side of the figure), the green box part, which was critical for the determination of PERV-A/C is enlarged. Blue boxes show the PERV-A/C cluster, whereas the red one shows the PERV-C cluster. The sequences of m190424_... are the NGS data.

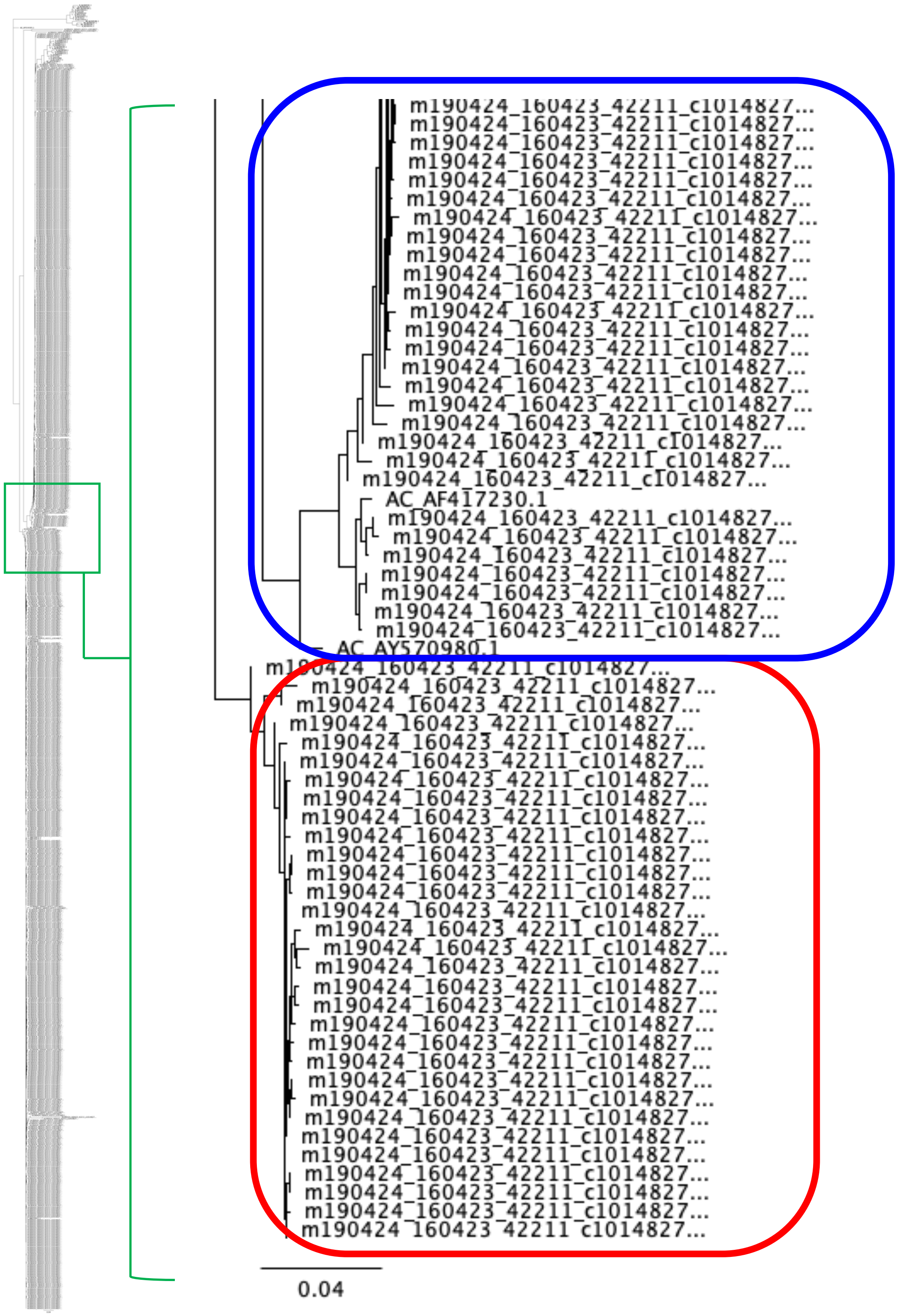

Figure S3. A representative phylogenetic tree of the NGS data of the 10 % PERV-A/C sample (Experimental ID: RSII 1). As there were too many reads to show all the data (left side of the figure), the green box part, which was critical for the determination of PERV-A/C is enlarged. Blue boxes show the PERV-A/C cluster, whereas the red one shows the PERV-C cluster. The sequences of m190424_... are the NGS data.

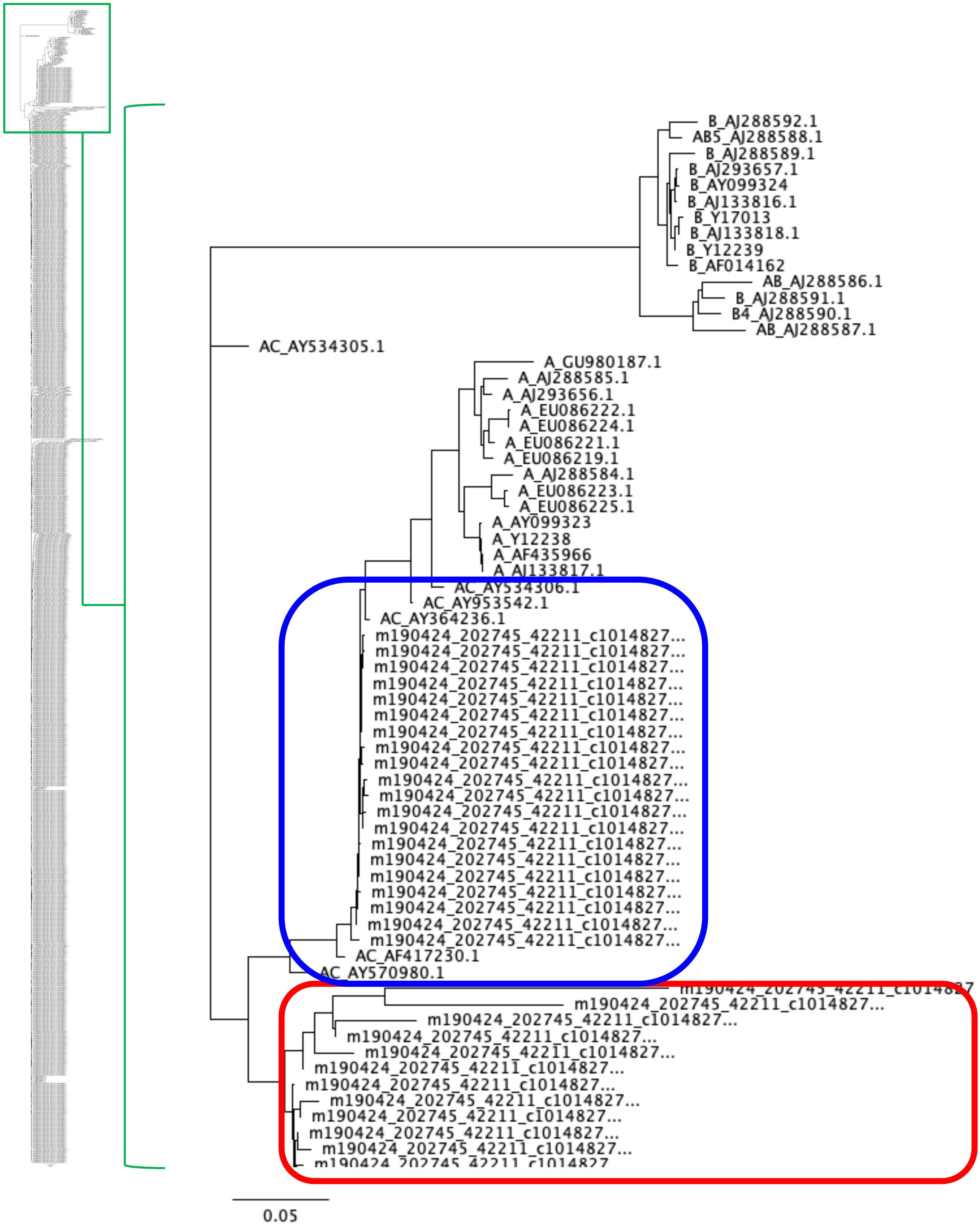

Figure S4. A representative phylogenetic tree of the NGS data of the 1 % PERV-A/C sample (Experimental ID: RSII 1). As there were too many reads to show all the data (left side of the figure), the green box part, which was critical for the determination of PERV-A/C is enlarged. The blue box shows the PERV-A/C cluster, whereas the red one shows the PERV-C cluster. The sequences of m190424_... are the NGS data.

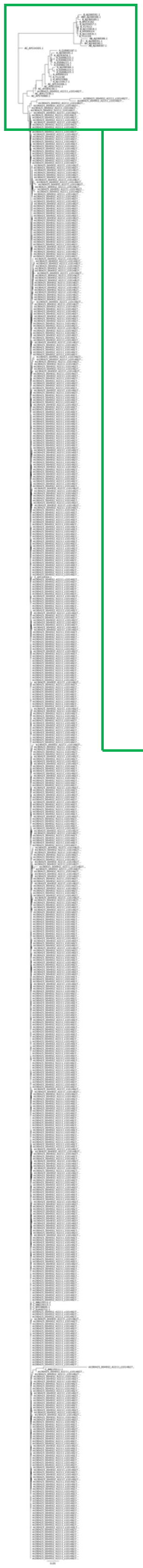

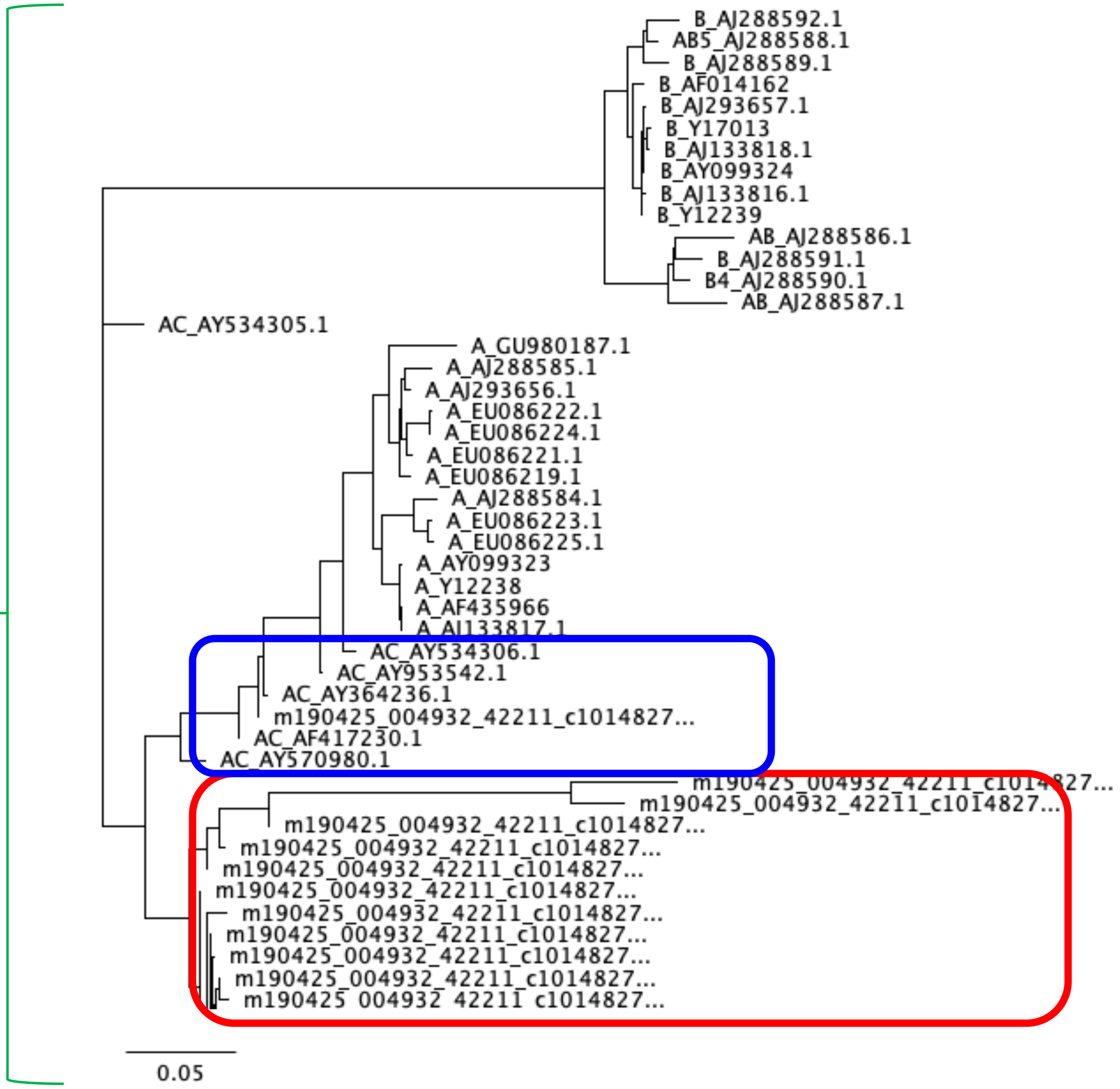

Figure S5. A representative phylogenetic tree of the NGS data of the 0.1 % PERV-A/C sample (Experimental ID: RSII 1). As there were too many reads to show all the data (left side of the figure), the green box part, which was critical for the determination of PERV-A/C is enlarged. Blue boxes show the PERV-A/C cluster, whereas the red one shows the PERV-C cluster. The sequences of m190425_... are the NGS data.

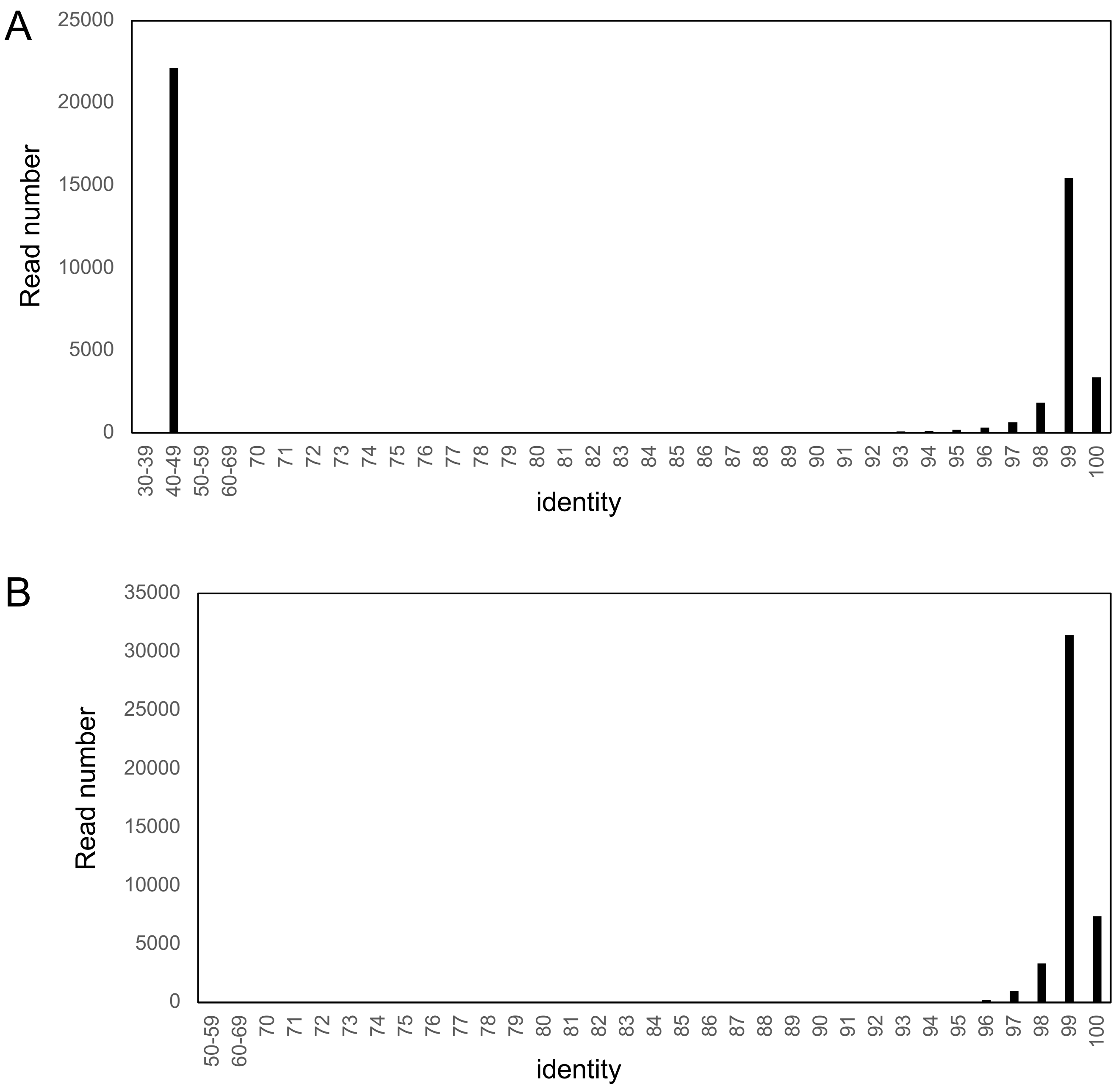

Figure S6. Comparison of identity distribution in the 0 % PERV-A/C (100 % PERV-C) sample. The identity refers to the similarity to the query sequence (PERV-C) and was calculated by the global (A) and local (B) alignments.

A

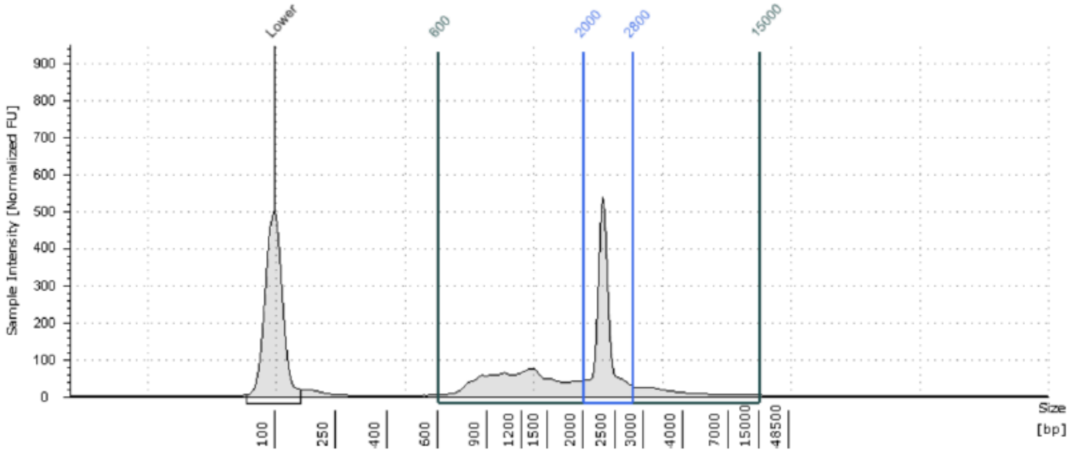

B

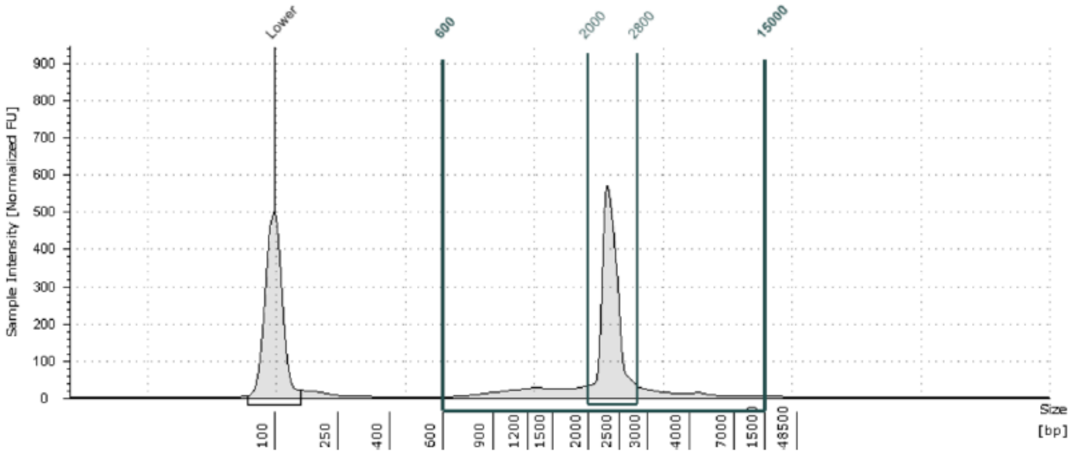

Figure S7. The purity of RT-PCR products applied for Amplicon-Seq analysis was measured using the Agilent 2100 Bioanalyzer. PERV-A/C recombinants were not detected in the low purified 1 % sample (Experimental ID: RSII 7) (A), whereas they were detected in the highly purified 0.1 % sample (Experimental ID: RSII 3) (B).

```
AC_AF417230.1.fasta       1 ATGCATCCCACGTTAAGCCGGCGCCACCTCCCGATTCGGGGTGGAAAGCCGAAAAGACTG     60
PERV-AC(13653).fasta      1 ............................................................     60

AC_AF417230.1.fasta      61 AAAATCCCCTTAAGCTTCGCCTCCATCGCGTGGTTCCTTACTCTGTCAATAACCTCTCAG    120
PERV-AC(13653).fasta     61 .....................................................TC....A    120

AC_AF417230.1.fasta     121 ACTAATGGTATGCGCATAGGAGACAGCCTGAACTCCCATAAACCCTTATCTCTCACCTGG    180
PERV-AC(13653).fasta    121 GT........AA...C.T.TG.......C...............................    180

AC_AF417230.1.fasta     181 TTAATTACTGACTCCGGTACAGGTATTAATATTAACAGCACTCAAGGGGAGGCTCCCTTG    240
PERV-AC(13653).fasta    181 ...C........................................................    240

AC_AF417230.1.fasta     241 GGGACCTGGTGGCCTGAATTATATGTCTGCCTTCGATCAGTAATCCCTGGTCTCAATGAC    300
PERV-AC(13653).fasta    241 ............................................................    300

AC_AF417230.1.fasta     301 CAGGCCACACCCCCCGATGTACTCCGTGCTTACGGGTTTTACGTTTGCCCAGGACCCCCA    360
PERV-AC(13653).fasta    301 ............................................................    360

AC_AF417230.1.fasta     361 AATAATGAAGAATATTGTGGAAATCCTCAGGATTTCTTTTGCAAGCAATGGAGCTGCGTA    420
PERV-AC(13653).fasta    361 .........................................................A..    420

AC_AF417230.1.fasta     421 ACTTCTAATGATGGGAATTGGAAATGGCCAGTCTCTCAGCAAGACAGAGTAAGTTACTCT    480
PERV-AC(13653).fasta    421 ............................................................    480

AC_AF417230.1.fasta     481 TTTGTTAACAATCCTACCAGTTATAATCAATTTAATTATGGCCATGGGAGATGGAAAGAT    540
PERV-AC(13653).fasta    481 ............................................................    540

AC_AF417230.1.fasta     541 TGGCAACAGCGGGTACAAAAAGATGTACGAAATAAGCAAATAAGCTGTCATTCGTTAGAC    600
PERV-AC(13653).fasta    541 ............................................................    600

AC_AF417230.1.fasta     601 CTAGATTACTTAAAAATAAGTTTCACTGAAAAAGGAAAACAAGAAAATATTCAAAAGTGG    660
PERV-AC(13653).fasta    601 ............................................................    660

AC_AF417230.1.fasta     661 GTAAATGGTATGTCTTGGGGAATAGTGTACTATGGAGGCTCTGGGAGAAAGAAAGGATCT    720
PERV-AC(13653).fasta    661 ..........A.................................................    720

AC_AF417230.1.fasta     721 GTTCTGACTATTCGCCTCAGAATAGAAACTCAGATGGAACCTCCGGTTGCTATAGGACCA    780
PERV-AC(13653).fasta    721 ............................................................    780

AC_AF417230.1.fasta     781 AATAAGGGTTGGGCCAAACAAGGACCTCCAATCCAAGAACAGAGGCCATCTCCTAACCCC    840
PERV-AC(13653).fasta    781 ..........T....G............................................    840

AC_AF417230.1.fasta     841 TCTGATTACAATACAACCTCTGGATCAGTCCCCACTGAGCCTAACATCACTATTAAAACA    900
PERV-AC(13653).fasta    841 ............................................................    900

AC_AF417230.1.fasta     901 GGGGCGAAACTTTTTAACCTCATCCAGGGAGCTTTTCAAGCTCTTAACTCCACAACTCCA    960
PERV-AC(13653).fasta    901 ................G....................................G......    960

AC_AF417230.1.fasta     961 GAGGCTACCTCTTCTTGTTGGCTTTGCTTAGCTTCGGGCCCACCTTACTATGAGGGAATG   1020
PERV-AC(13653).fasta    961 ............................................................   1020

AC_AF417230.1.fasta    1021 GCTAGAGGAGGGAAATTCAATGTGACAAAGGAACATAGAGACCAATGTGCATGGGGATCC   1080
PERV-AC(13653).fasta   1021 ................................................A...........   1080

AC_AF417230.1.fasta    1081 CAAAATAAGCTTACCCTTACTGAGGTTTCTGGAAAAGGCACCTGCATAGGAAAGGTTCCC   1140
PERV-AC(13653).fasta   1081 ..................................................G.........   1140

AC_AF417230.1.fasta    1141 CCATCCCACCAACACCTTTGTAACCACACTGAAGCCTTTAATCAAACCTCTGAGAGTCAA   1200
PERV-AC(13653).fasta   1141 ............................................................   1200

AC_AF417230.1.fasta    1201 TATCTGGTACCTGGTTATGACAGGTGGTGGGCATGTAATACTGGATTAACCCCTTGTGTT   1260
PERV-AC(13653).fasta   1201 ............................................................   1260

AC_AF417230.1.fasta    1261 TCCACTTTGGTTTTTAACCAAACTAAAGATTTTTGCATTATGGTCCAAATTGTTCCCCGA   1320
PERV-AC(13653).fasta   1261 .....C......................................................   1320

AC_AF417230.1.fasta    1321 GTGTATTACTATCCCGAAAAAGCAATCCTTGATGAATATGACTACAGAAATCATCGACAA   1380
PERV-AC(13653).fasta   1321 ............................................................   1380

AC_AF417230.1.fasta    1381 AAGAGAGAACCCATATCTCTGACACTTGCTGTGATGCTCGGACTTGGAGTGGCAGCAGGT   1440
PERV-AC(13653).fasta   1381 ............................................................   1440

AC_AF417230.1.fasta    1441 GTAGGAACAGGAACAGCTGCCCTGGTCACGGGACCACAGCAGCTAGAAACAGGACTTAGT   1500
PERV-AC(13653).fasta   1441 ............................................................   1500

AC_AF417230.1.fasta    1501 AACCTACATCGAATTGTAACAGAAGATCTCCAAGCCCTAGAAAAATCTGTCAGTAACCTG   1560
PERV-AC(13653).fasta   1501 ............................................................   1560

AC_AF417230.1.fasta    1561 GAGGAATCCCTAACCTCCTTATCTGAAGTAGTCCTACAGAATAGAAGAGGGTTAGATTTA   1620
PERV-AC(13653).fasta   1561 ............................................................   1620

AC_AF417230.1.fasta    1621 TTATTTCTAAAAGAAGGAGGATTATGTGTAGCCTTGAAGGAGGAATGCTGTTTTTATGTG   1680
PERV-AC(13653).fasta   1621 ............................................................   1680

AC_AF417230.1.fasta    1681 GATCATTCAGGGGCCATCAGAGACTCCATGAACAAGCTTAGAGAAAGGTTGGAGAAGCGT   1740
PERV-AC(13653).fasta   1681 ...................................A........................   1740

AC_AF417230.1.fasta    1741 CGAAGGGAAAAGGAAACTACTCAAGGGTGGTTTGAGGGATGGTTCAACAGGTCTCCTTGG   1800
PERV-AC(13653).fasta   1741 ............................................................   1800

AC_AF417230.1.fasta    1801 TTGGCTACCCTACTTTCTGCTTTAACAGGACCCTTAATAGTCCTCCTCCTGTTACTCACA   1860
PERV-AC(13653).fasta   1801 ............................................................   1860

AC_AF417230.1.fasta    1861 GTTGGGCCATGTATTATTAACAAGTTAATTGCCTTCATTAGAGAACGAATAAGTGCAGTC   1920
PERV-AC(13653).fasta   1861 ............................................................   1920

AC_AF417230.1.fasta    1921 CAGATCATGGTACTTAGACAACAGTACCAAAGCCCGTCTAGCAGGGAAGCTGGCCGCTAG   1980
PERV-AC(13653).fasta   1921 ............................................................   1980
```

Figure S8. Alignments of *env* sequences of PERV-A/C (AF417230.1) and PERV-A/C (13653) was generated in this study. Dots denote the nucleotides identical to that of PERV-A/C (AF417230.1). The percent identity between the sequences was 98.8% (1957/1980).

Table S1 Summary of the number of reads from the NGS analysis of low purified samples

Exp ID	A/C:C Ratio (%)	total	over1500	Global identity 80-94	Global AC Reads	Local identity 70-94	Local AC Reads
RSII 7	0%	25,471	13,581	309	0	689	0
	10%	25,038	16,060	359	>100	861	>100
	1%	19,058	11,085	195	0	448	0
	0.1%	28,540	19,261	252	0	606	0
